# Differential regulation of extracellular matrix protein expression in carcinoma-associated fibroblasts by TGF-β1 regulates cancer cell spreading but not adhesion

**DOI:** 10.18632/oncoscience.87

**Published:** 2014-10-15

**Authors:** Mieke Van Bockstal, Kathleen Lambein, Mireille Van Gele, Elly De Vlieghere, Ridha Limame, Geert Braems, Rudy Van den Broecke, Veronique Cocquyt, Hannelore Denys, Marc Bracke, Louis Libbrecht, Olivier De Wever

**Affiliations:** ^1^ Department of Pathology, Ghent University and Ghent University Hospital, Ghent, Belgium; ^2^ Department of Dermatology, Ghent University Hospital, Ghent, Belgium; ^3^ Department of Radiation Oncology and Experimental Cancer Research, Ghent University and Ghent University Hospital, Ghent, Belgium; ^4^ Department of Gynaecology, Ghent University Hospital, Ghent, Belgium; ^5^ Department of Medical Oncology, Ghent University Hospital, Ghent, Belgium

**Keywords:** Cancer-associated fibroblasts Stromal protein expression TGF-β1 Decorin Versican

## Abstract

Cancer progression is characterized by a complex reciprocity between neoplastic epithelium and adjacent stromal cells. In ductal carcinoma in situ (DCIS) of the breast, both reduced stromal decorin expression and myxoid stroma are correlated with increased recurrence risk. In this study, we aimed to investigate paracrine regulation of expression of decorin and related extracellular matrix (ECM) proteins in cancer-associated fibroblasts (CAFs). Transforming growth factor-β1 (TGF-β1) was identified as a competent ECM modulator, as it reduced decorin and strongly enhanced versican, biglycan and type I collagen expression. Similar but less pronounced effects were observed when fibroblasts were treated with basic fibroblast growth factor (bFGF). Despite this concerted ECM modulation, TGF-β1 and bFGF differentially regulated alpha-smooth muscle actin (α-SMA) expression, which is often proposed as a CAF-marker. Cancer cell-derived secretomes induced versican and biglycan expression in fibroblasts. Immunohistochemistry on twenty DCIS specimens showed a trend toward periductal versican overexpression in DCIS with myxoid stroma. Cancer cell adhesion was inhibited by decorin, but not by CAF-derived matrices. Cancer cells presented significantly enhanced spreading when seeded on matrices derived from TGF-β1-treated CAF. Altogether these data indicate that preinvasive cancerous lesions might modulate the composition of surrounding stroma through TGF-β1 release to obtain an invasion-permissive microenvironment.

## INTRODUCTION

The significance of the tumor microenvironment in cancer progression has been increasingly acknowledged. Cancer progression is the result of complex cross-talk between neoplastic cells and neighbouring stromal cells, such as fibroblasts, endothelial cells, adipocytes and inflammatory cells [1, 2]. Gene expression profiling of neoplastic epithelium and stroma during breast cancer progression revealed that the expression of molecules involved in extracellular matrix (ECM) remodeling is altered during the transition from ductal carcinoma in situ (DCIS) to invasive ductal carcinoma (IDC) [3-6]. Extensive stromal gene expression alterations also occur during the transition from normal breast to DCIS [4]. Gevaert et al. reported overexpression of the small leucin-rich proteoglycans (SLRP) decorin and fibromodulin in a gene expression signature associated with good prognosis in invasive breast cancer [7]. Decreased stromal decorin and lumican correlated with worse prognosis in lymph node-negative invasive breast cancer [8]. Immunohistochemical (IHC) analysis showed that stromal decorin expression is highest in normal breast tissue, lower in DCIS and lowest in IDC [9]. A similar trend was noticed in the colon, with strong decorin expression in normal tissue, hyperplastic adenomas and the majority of tubular adenomas, and decreased decorin expression in tubulo-villous adenomas and most adenocarcinomas [10].

We recently observed that decreased periductal decorin immunoreactivity correlated with the presence of myxoid stromal architecture in DCIS. Reduced stromal decorin and myxoid stroma were both significantly associated with an increased recurrence risk in DCIS [11]. As not all DCIS will evolve to IDC, stromal alterations might enable the identification of lesions that possess the capability to progress. We hypothesized that decreased stromal decorin may contribute to the pathogenesis of myxoid stroma, as decorin seems to play an important role in collagen fibrillogenesis [12]. Myxoid stroma and the associated decorin reduction might mirror the propensity of some DCIS lesions to progress to IDC. Neoplastic lesions may induce ECM alterations through paracrine corruption of fibroblasts, resulting in an invasion-permissive stroma. In this study, we aimed to investigate paracrine regulation of stromal decorin expression, as well as related ECM protein expression. Immortalized CAF were used as an *in vitro* model. *In vitro* findings were translated to the clinical setting by performing IHC on DCIS specimens. In addition, we investigated the effect of decorin and CAF-derived matrices on cancer cell adhesion. The main goal of this study was to gain more insight in cancer-induced changes in the peritumoral ECM, since this might aid the identification of new therapeutic targets, as well as novel prognostic markers.

## RESULTS

### TGF-β1 and bFGF suppress decorin expression at the protein level

**Figure 1:**
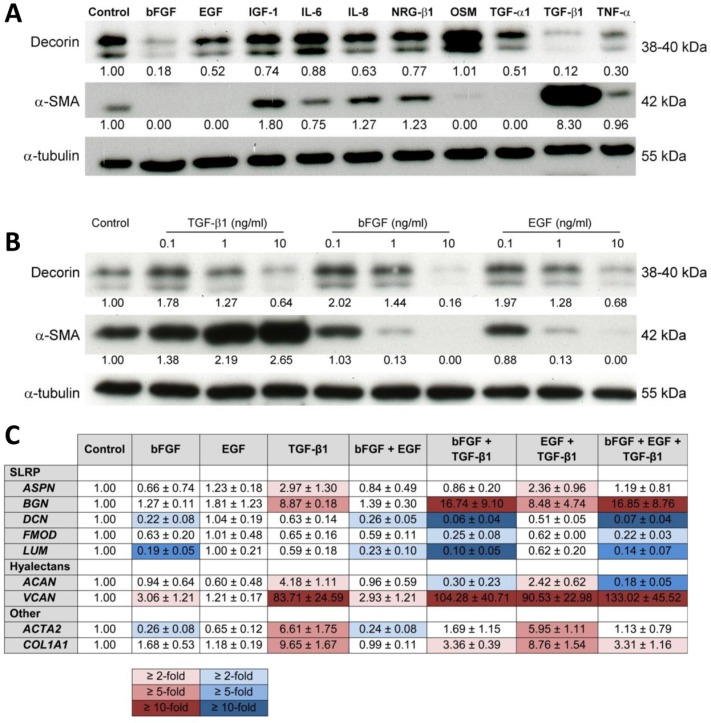
Cytokine treatment influences ECM protein expression in CAFs (A) Western blot showing the effect of treatment of CAFs with ten different growth factors (all at a concentration of 10 ng/ml) on the expression of decorin and α-SMA. Tubulin was used as a loading control. (B) Western blot illustrating the effect of three different concentrations of TGF-β1, bFGF or EGF on the expression of decorin and α-SMA in CAFs. Tubulin was used as a loading control. (C) Table displaying the normalized mRNA expression profile of five SLRP's, two hyalectans, type 1 collagen and α-SMA in CAFs after treatment with bFGF, EGF or TGF-β1, either alone or in combination (10 ng/ml). Red and blue color scales represent 2-, 5- and 10-fold up- and downregulation, respectively. Values are mean ± SD and represent 3 independent experiments.

We screened ten cytokines for the differential regulation of decorin expression in CAFs. TGF-β1and bFGF were the strongest suppressors of decorin expression, followed by TNF-α, TGF-α1 and EGF (Figure 1A). TGF-β1 and bFGF treatment resulted in 10-fold and 5-fold downregulation, respectively. Additionally, we assessed the effect of these cytokines on α-SMA expression, as this molecule is often presented as a CAF-marker [13-15]. Expression of α-SMA in CAFs was 8-fold upregulated by TGF-β1, whereas bFGF, EGF and TGF-α1 completely suppressed α-SMA expression, as previously observed [15, 16]. The differential regulation of α-SMA and decorin prompted us to select bFGF, EGF and TGF-β1 for further experiments. Next, CAFs were treated with bFGF, EGF and TGF-β1 in three concentrations. Downregulation of decorin expression was only observed when cells were treated with the highest concentration of 10 ng/ml (Figure 1B). A concentration of 1 ng/ml bFGF, EGF or TGF-β1 exerted distinct effects on α-SMA expression, and these were magnified by increasing the concentration to 10 ng/ml. Therefore, we selected the latter for subsequent experiments.

### TGF-β1 and bFGF differentially modulate ECM protein expression

CAFs were treated with bFGF, EGF and TGF-β1 at 10 ng/ml, either alone or in combination. RT-qPCR was performed to assess the mRNA expression of *ACTA2*, *COL1A1*, five SLRP's (*ASPN*, *BGN*, *DCN*, *FMOD*, *LUM*) and two hyalectans (*VCAN* and *ACAN*). EGF did not exert significant effects on mRNA expression (Figure 1C). *ACTA2* was about 4-fold downregulated by bFGF and >6-fold upregulated by TGF-β1. When bFGF and TGF-β1 were combined, the stimulatory TGF-β1 effect was almost completely counteracted by bFGF, while EGF was not able to neutralize this TGF-β1 effect (Figure 1C).

**Figure 2:**
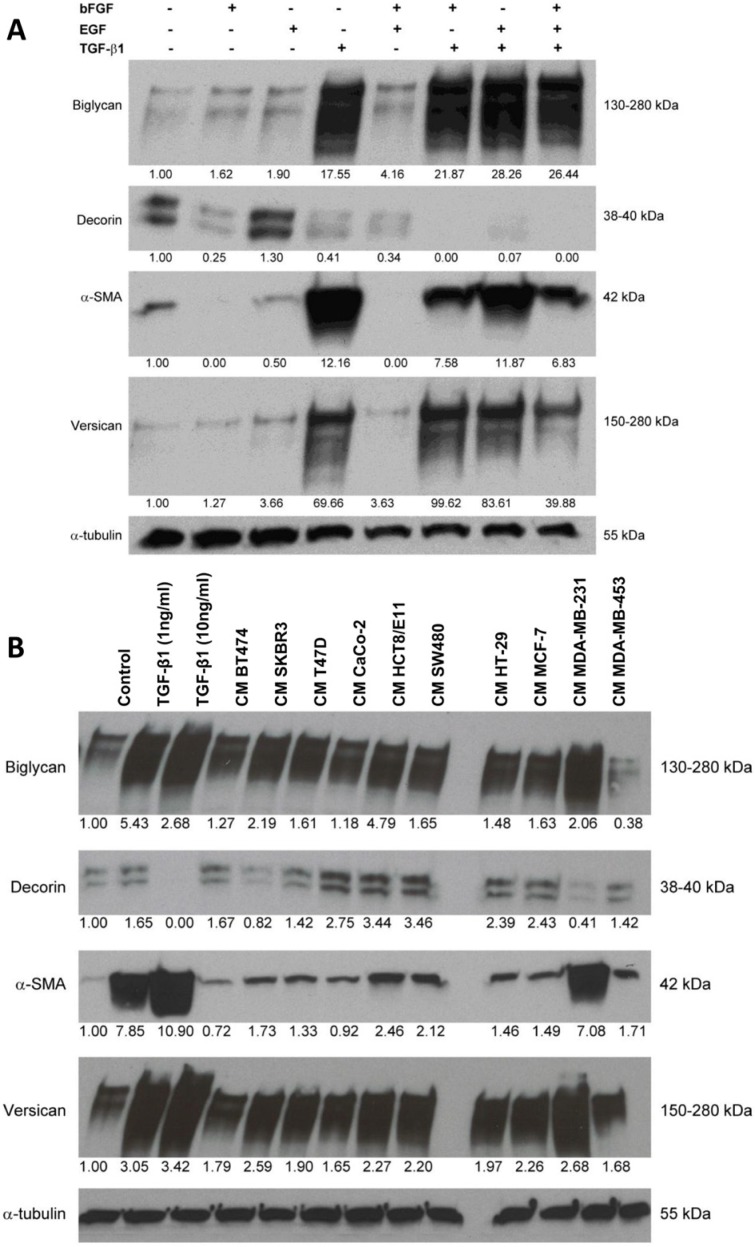
Treatment of CAFs with combined cytokines or cancer cell-derived secretomes affects ECM protein expression (A) Western blot showing the effects of bFGF, EGF and TGF-β1 treatment (10 ng/ml) on the expression of biglycan, decorin, α-SMA and versican in CAFs. (B) Western blot illustrating the effects of treatment with cancer cell-derived conditioned medium (CM) on the expression of biglycan, decorin, α-SMA and versican in CAFs. TGF-β1 treatment (1 and 10 ng/ml) was included as a positive control. Tubulin was used as loading control (A, B).

*BGN* and *VCAN* displayed a similar mRNA expression pattern: both were strongly induced by TGF-β1, and this was enhanced by bFGF, resulting in a >15-fold upregulation of *BGN* and a >100-fold upregulation of *VCAN* (Figure 1C). *COL1A1* expression was almost 10-fold induced by TGF-β1, though this was attenuated by bFGF. *ASPN* expression was 3-fold stimulated by TGF-β1, but bFGF did not significantly influence its expression. Three other SLRP's were expressed alike: *DCN*, *FMOD* and *LUM* were all downregulated by bFGF and this effect was enhanced by TGF-β1. *ACAN*, a hyalectan, was 4-fold upregulated by TGF-β1 but this effect was attenuated by EGF, whereas the combination of bFGF and TGF-β1 caused a 3-fold downregulation. Altogether, these findings point to differential regulation of ECM proteins, with upregulation of versican, biglycan, *COL1A1* and α-SMA as a signature of TGF-β1-induced protein expression. This experiment was repeated to confirm the aforementioned RT-qPCR results at the protein level by Western blotting. TGF-β1 strongly induced versican, biglycan and α-SMA, and caused downregulation of decorin in fibroblasts. Similar to the RT-qPCR findings, bFGF treatment only had a moderate effect on the upregulation of versican and biglycan, whereas it effectively suppressed decorin and α-SMA expression. When bFGF was combined with TGF-β1, the stimulatory TGF-β1 effect on α-SMA expression was clearly attenuated, whereas decorin downregulation and biglycan and versican upregulation were enhanced (Figure 2A).

### Cancer cell-derived secretomes affect ECM protein expression in CAFs

Conditioned medium (CM) was collected from six breast cancer cell lines and four colorectal cancer cell lines. CAFs were treated with this CM for 6 days. Fibroblasts treated with TGF-β1 (1 and 10 ng/ml) were included as a positive control (Figure 2B). Although CM of almost every cancer cell line caused a slight to moderate upregulation of α-SMA, CM of MDA-MB-231 caused a distinct increase in α-SMA expression. Simultaneously, it was the most potent suppressor of decorin expression and the strongest inducer of biglycan and versican. CM of all breast and colorectal cancer cell lines enhanced versican and biglycan expression. SKBR3 was the only other breast cancer cell line of which CM downregulated decorin. Remarkably, CM of all colorectal cancer cell lines caused increased decorin expression, but presently we cannot explain this differential regulation.

### Immunohistochemical evaluation of ECM protein expression

**Figure 3:**
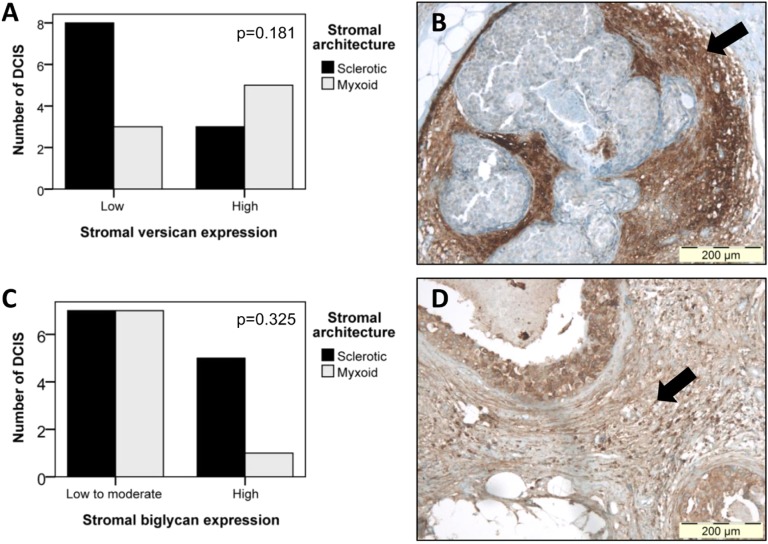
Comparison of stromal versican and biglycan expression in DCIS lesions with sclerotic or myxoid stroma (A) Bar chart displaying the relation between stromal versican expression and stromal architecture. Despite a trend toward high versican expression in DCIS with myxoid stroma, statistical significance was not reached (p=0.181), due to lack of power (β=0.803). (B) Photomicrograph displaying high periductal versican immunoreactivity (arrow) in a DCIS lesion with myxoid stroma. Original magnification 100x. (C) Bar chart illustrating the absence of any association (p=0.325) between stromal biglycan expression and stromal architecture. (D) Photomicrograph illustrating high periductal biglycan staining (arrow) in a DCIS lesion with myxoid stroma. Original magnification 100x.

**Figure 4:**
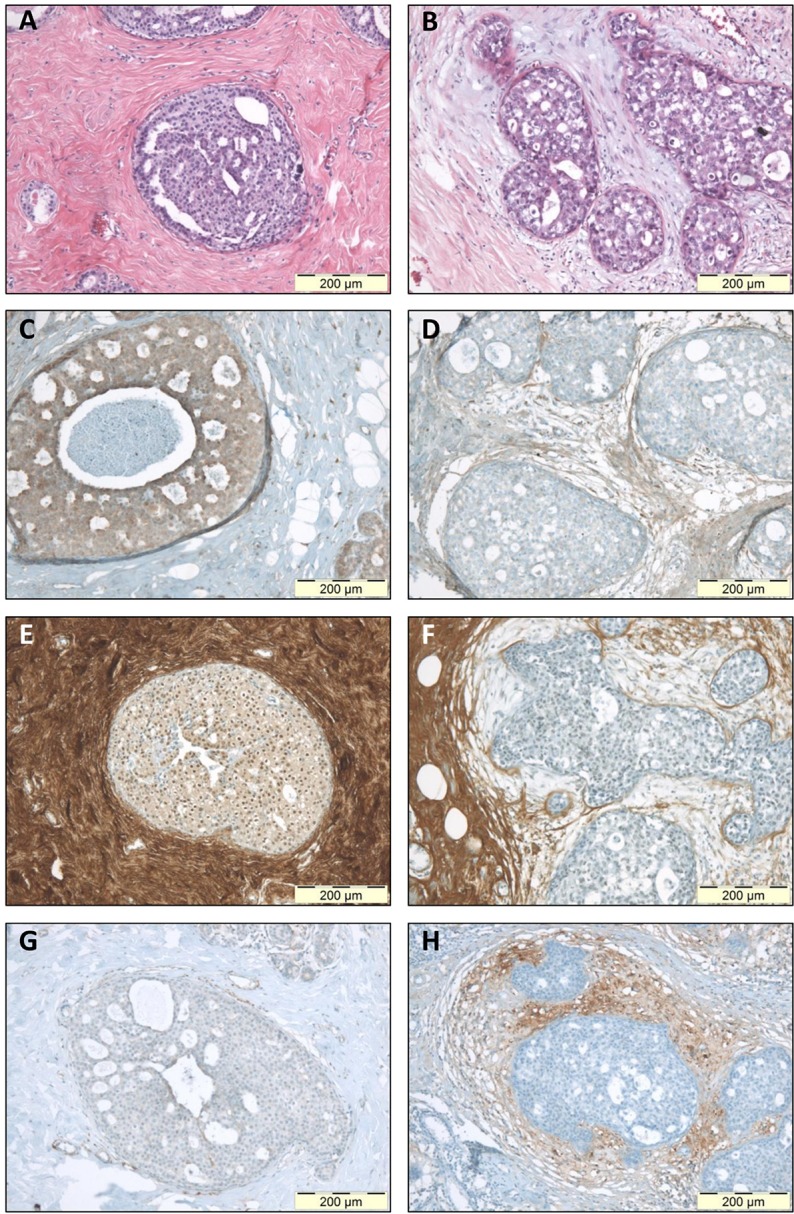
Immunohistochemical staining of stromal protein expression in DCIS with sclerotic or myxoid stroma Microphotographs displaying HE staining (A-B), and IHC staining for biglycan (C-D), decorin (E-F) and versican (G-H). Panels A-C-E-G display photographs of one DCIS lesion with sclerotic stroma; panels B-D-F-H display one DCIS lesion with myxoid stroma. This figure illustrates that myxoid DCIS present reduced periductal decorin staining and tend to have increased periductal versican and biglycan expression, whereas sclerotic DCIS generally present strong stromal decorin immunoreactivity, and tend to lack stromal versican and biglycan. Original magnification 100x.

Since we previously reported a significant association between periductal myxoid stroma and reduced stromal decorin in DCIS [11], we aimed to investigate whether a correlation existed between myxoid stroma and increased biglycan and versican immunoreactivity. IHC was performed on eight myxoid DCIS, and twelve DCIS with sclerotic stroma. In DCIS with low stromal versican, 3 of 11 (27%) lesions presented myxoid stroma, while DCIS with high stromal versican counted 5 of 8 (63%) myxoid cases (Figure 3A-B). Despite this trend, statistical significance was not reached (p=0.181), which is likely due to lack of power. Calculated power amounted only 0.197 (β=0.803). Stromal biglycan expression was not able to discern myxoid from sclerotic DCIS (Figure 3C-D): in DCIS with low to moderate stromal biglycan, 7 of 14 (50%) DCIS presented myxoid stroma, while 1 of 6 DCIS (17%) with high stromal biglycan had myxoid stroma (p=0.325) (Figure 4).

### Decorin negatively influences cancer cell adhesion

In addition to paracrine regulation of stromal protein expression, we aimed to investigate the effects of decorin on cancer cell adhesion. Human recombinant His-tagged decorin was purified from CM of decorin-overexpressing HEK-293-EBNA-*DCN* cells. Western blotting confirmed purification of both His-tagged core protein (50 kDa) and glycosylated decorin (75-160 kDa). As a quality control, purified decorin was incubated with chondroitinase ABC, a chondroitin and dermatan sulphate degrading enzyme [17]. After digestion, Western blotting showed that only His-tagged core protein remained (results not shown).

**Figure 5:**
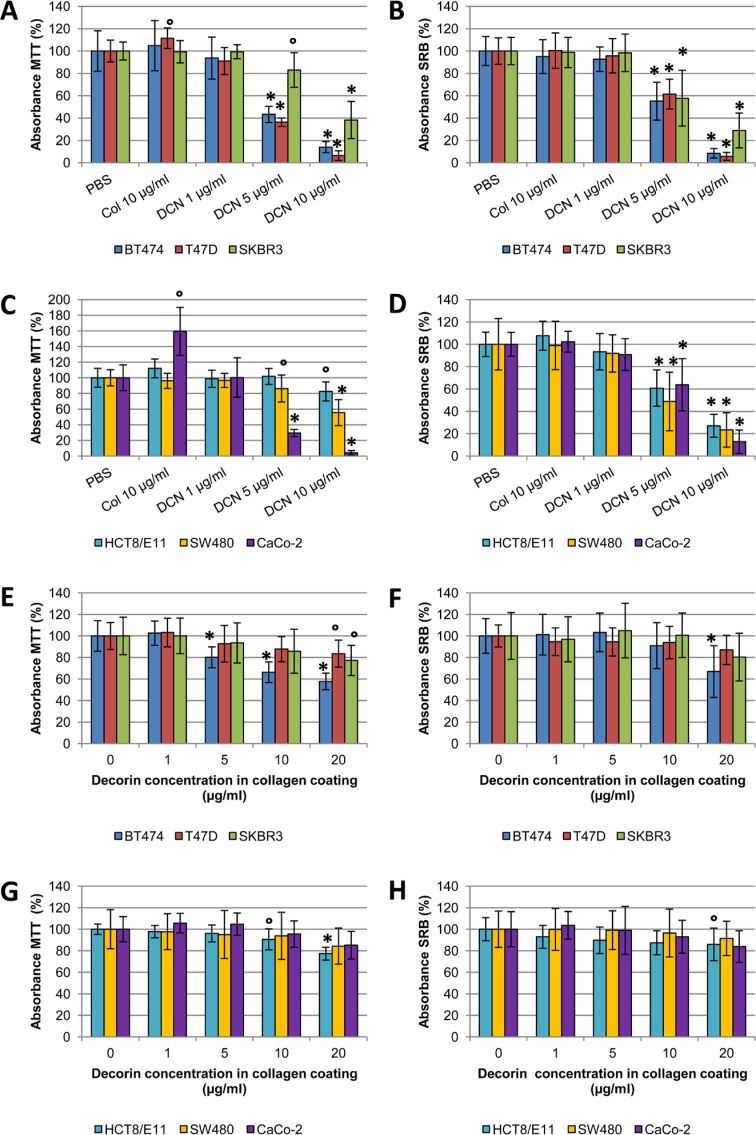
SRB and MTT adhesion assays with cancer cells seeded onto coatings containing type I collagen and decorin (A-D) Bar charts displaying the effect of pure decorin coatings and a type I collagen coating on cancer cell adhesion, as measured by MTT (A,C) and SRB (B,D) assays. (E-H) Bar charts illustrating the effect of different concentrations of decorin on cancer cell adhesion when added to a type I collagen coating (10 μg/ml in PBS), as measured by MTT (E,G) and SRB (F,H) assays. All experiments were performed with three breast and three colorectal cancer cell lines, to exclude a cell line specific effect. Results are presented from five wells per assay from three independent experiments. Values are mean percentage ± SD. ° p<0.05 and * p<0.001 as compared with control. Col: type I collagen; DCN: decorin.

Three colorectal cancer cell lines (CaCo-2, HCT8/ E11 and SW480) and three breast cancer cell lines (BT474, SKBR3 and T47D) were seeded onto coatings of purified decorin in different concentrations, or type I collagen (10 μg/ml in PBS). Multiple cell lines were used to exclude a cell line specific effect. Experiments were performed in duplicate, to quantify adhesion by SRB staining and to assess effects of the coatings on cell metabolism by MTT assays. Collagen coatings did not affect cell adhesion as compared with PBS control (Figure 5A-D), but significantly increased metabolic activity in CaCo-2 cells was observed (p=0.015). Decorin coatings inhibited attachment of all cell lines in a dose-dependent manner. This anti-adhesive effect was statistically significant when a concentration of 5 μg/ml decorin was used (p≤0.007 for all cell lines) and was even more distinct with 10 μg/ml decorin (p≤0.002 for all cell lines). Altogether, these findings indicate that cancer cells attach to type I collagen, but they are not able to attach to decorin as a single substrate.

We wondered whether decorin exerted anti-adhesive effects in the presence of type I collagen. Experiments were repeated with combined coatings, containing 10 μg/ ml type I collagen and varying amounts of purified decorin (range 1-20 μg/ml). When cell adhesion was assessed by SRB assay, BT474 and HCT8/E11 were the only cell lines of which adhesion was significantly decreased when 20 μg/ml decorin was added to the coating (p<0.001 and p=0.030, respectively). BT474 was the only cancer cell line of which the metabolism significantly diminished when 5 μg/ml decorin or more was added to the type I collagen coating (p<0.001). The highest decorin concentration of 20 μg/ml also decreased cell metabolism in SKBR3 (p=0.010), T47D (p=0.020), CaCo-2 (p=0.009) and HCT8/E11 (p<0.001). Overall, type I collagen clearly counteracted the anti-adhesive effect of decorin (Figure 5 E-H).

**Figure 6:**
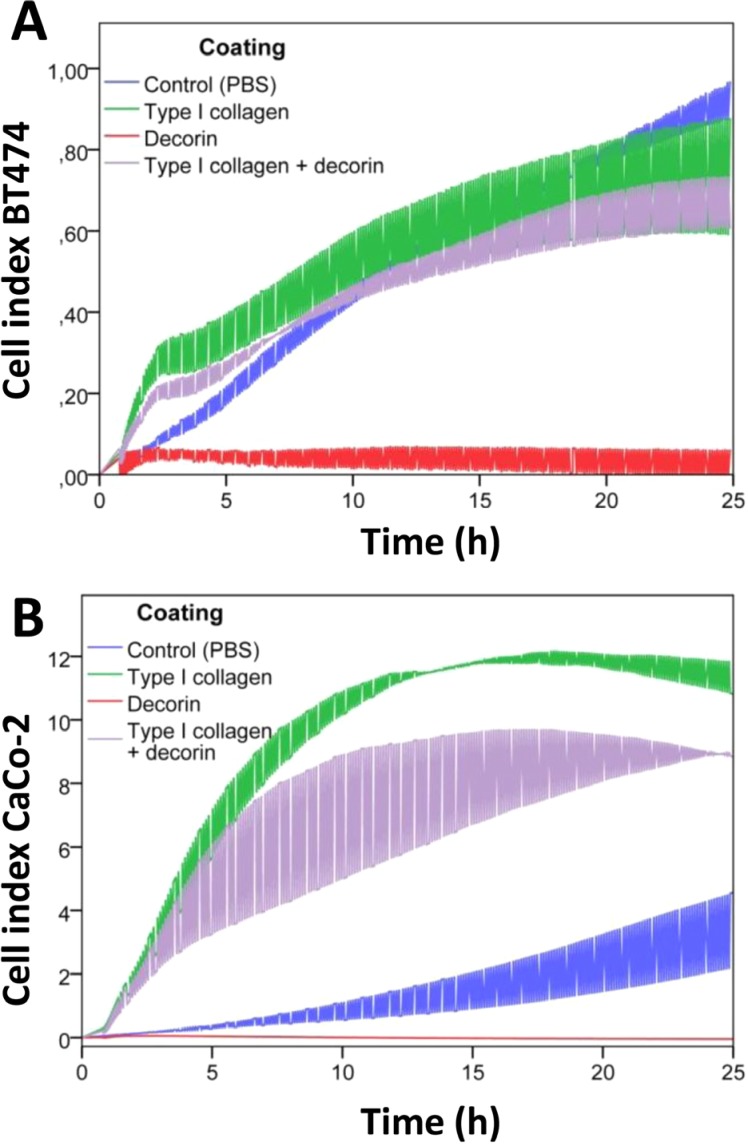
Real-time monitoring of cancer cell adhesion on decorin and type I collagen coatings Real-time monitoring of cell adhesion of BT474 (A) and CaCo-2 (B) cells by measuring electrical impedance during 24h. The cell index, i.e. the change in electrical impedance, is displayed in function of time (in hours) as mean ± SD. Cells were seeded on coatings containing PBS only (blue), type I collagen (green), decorin (red) or type I collagen combined with decorin (purple).

In addition, cell adhesion was assessed by an electrical impedance assay (Figure 6). BT474 and CaCo-2 cells were seeded onto coatings containing decorin, type I collagen or both. Decorin significantly inhibited adhesion in these cell lines (p=0.001 for both) compared with control coatings. Pure type I collagen coatings significantly enhanced adhesion of CaCo-2 cells compared with control coatings (p=0.001) or collagen/decorin coatings (p=0.021). Type I collagen coatings exerted an early stimulatory effect on adhesion of BT474 cells compared with control coatings, which caused a significant difference between the slopes of both curves (p=0.001). Addition of decorin to the type I collagen coating slightly diminished the cell index of BT474 cells, but did not affect the slope of the curve (p=0.834).

### CAF-derived matrices

**Figure 7:**
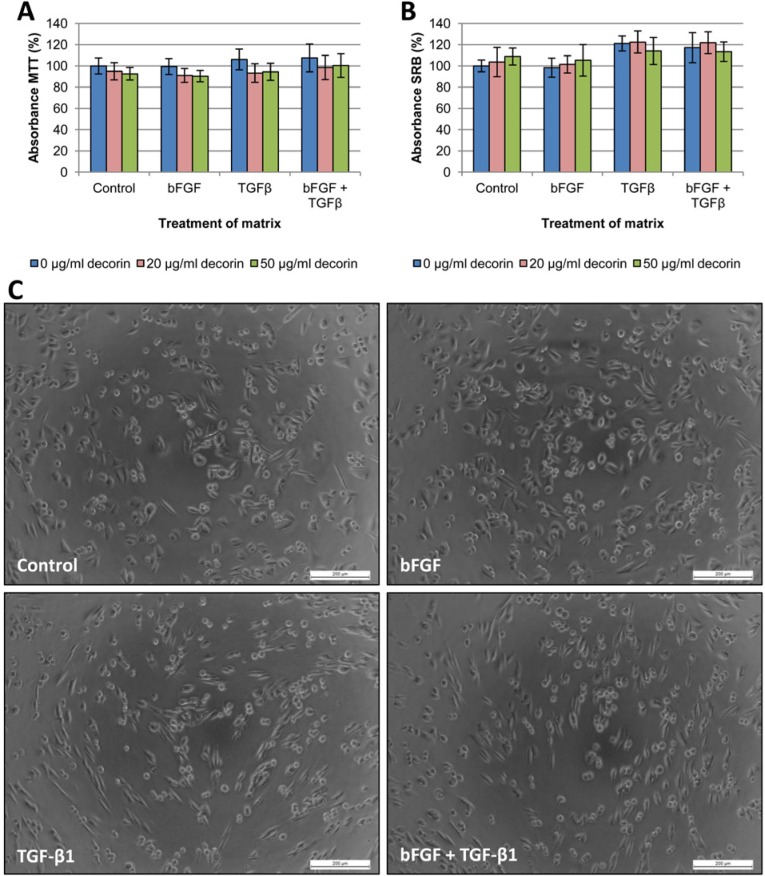
Adhesion assays with SW480 cells seeded onto CAF-derived matrices SW480 cells were seeded onto CAF-derived matrices with or without decorin coating, and cell adhesion was measured by MTT (A) and SRB (B) assay after 24 hours. Results are presented from three wells per assay from two independent experiments. Values are mean percentage ± SD. CAFs that produced the matrices were treated either with vehicle control, bFGF, TGF-β1 or both cytokines. (C) When seeded on matrices derived from TGF-β1-treated CAFs, elongated SW480 cells adhered in a highly organized fashion along preformed ‘tracks’ in the matrices, whereas the control or bFGF treatment showed a rather random distribution of the cells. Scale bar: 200 μm.

**Figure 8:**
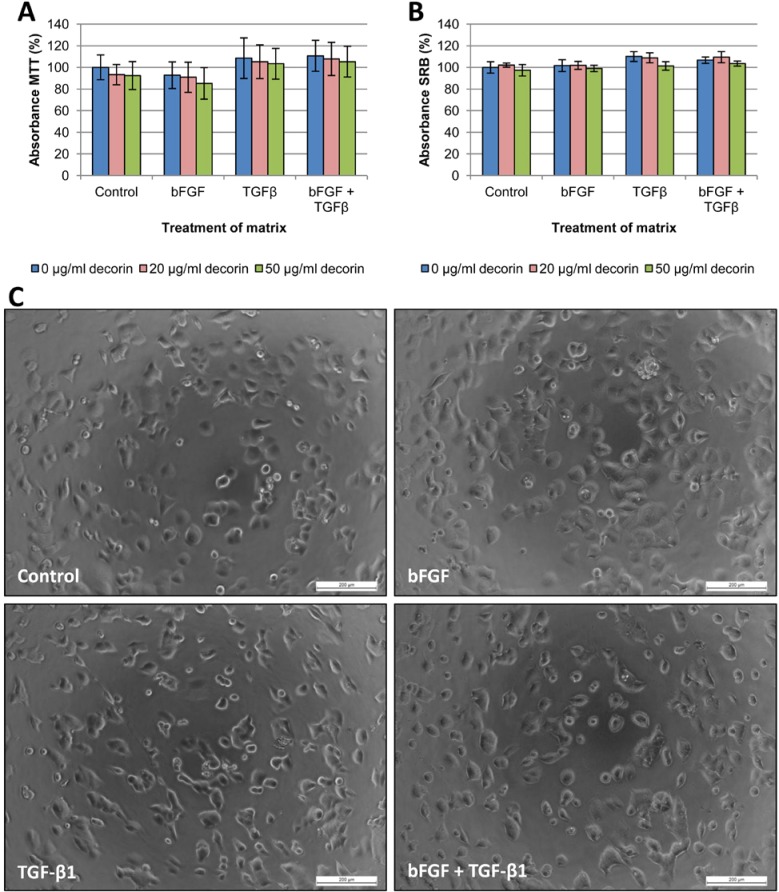
Adhesion assays with T47D cells seeded onto CAF-derived matrices T47D cells were seeded onto CAF-derived matrices with or without decorin coating, and cell adhesion was measured by MTT (A) and SRB (B) assay after 24 hours. Results are presented from three wells per assay from two independent experiments. Values are mean percentage ± SD. CAFs that produced the matrices were treated either with vehicle control, bFGF, TGF-β1 or both cytokines. (C) When seeded on matrices derived from TGF-β1-treated CAFs, T47D cells adhered in an organized fashion along preformed ‘tracks’ in the matrices, whereas the control or bFGF treatment showed a rather random distribution of the cells, with formation of more and larger cellular islands. Scale bar: 200 μm.

In addition to adhesion assays on single or dual reagent coatings, we attempted to mimic the *in vivo* situation by seeding cancer cells on CAF-derived matrices. Matrices were prepared as described by Castello-Cros et al., [18] and CAFs were treated six days with bFGF, TGF-β1 or both. After CAF removal, the remaining matrices were coated with decorin (20 or 50 μg/ml) or PBS control. Cancer cells were seeded onto matrices with or without decorin coating, and MTT and SRB adhesion assays were carried out after 24h incubation. These adhesion assays revealed no significant differences in cell adhesion (Figures 7-8). Moreover, application of decorin coatings on the matrices did not significantly inhibit adhesion, which is in line with the adhesion assays with combined coatings, where type I collagen counteracted the anti-adhesive effect of decorin. As CAFs produce type I collagen [19], a sufficient amount was present to neutralize the applied decorin coating.

However, phase-contrast microscopic analysis revealed morphological differences in the way cancer cells adhered to different matrices. SW480 cells, representative for colorectal cancer cells, and T47D, representative for breast cancer cells, are shown in Figure 7C and 8C, respectively. Cancer cells seeded on matrices derived from TGF-β1-treated fibroblasts seemed to attach along preformed ‘tracks’, compared with the random adhesion pattern of cells seeded on matrices from untreated or bFGF-treated CAFs. This phenomenon was most distinct with SW480 cells, which was probably due to their mesenchymal aspect: most SW480 cells are already spindle-shaped and therefore their uniform orientation was more obvious. We excluded that this phenomenon was due to TGF-β1 remnants in the matrices, by seeding TGF-β1-treated (1 μg/ml) cancer cells on matrices from untreated fibroblasts. All cell lines adhered in the same random pattern as cancer cells seeded on control matrices without TGF-β1 (results not shown).

**Figure 9:**
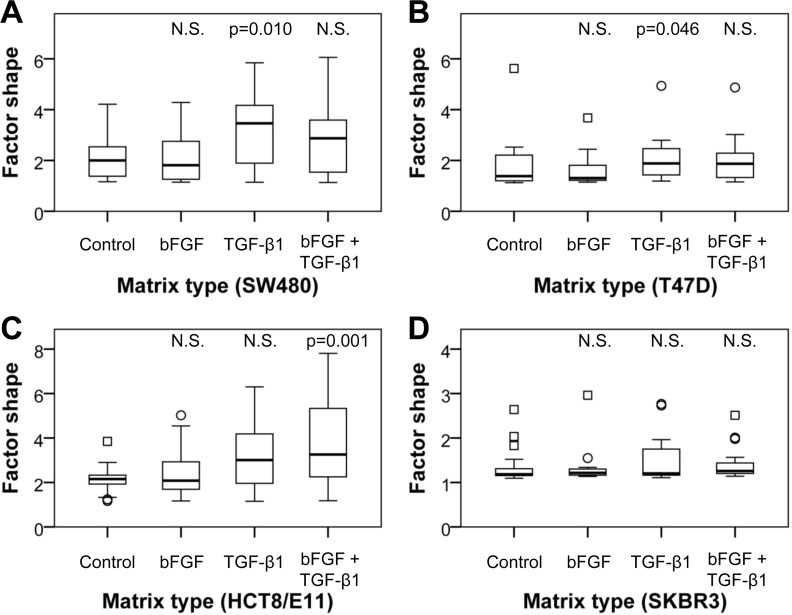
Quantification of cancer cell spreading on CAF-derived matrices Box plots illustrating the extent of cell spreading of SW480 (A), T47D (B), HCT8/E11 (C) and SKBR3 (D) cells on matrices as quantified by factor shape (= inverse circularity or P^2^/4πA). Matrices are derived from untreated CAFs and CAFs treated with bFGF, TGF-β1 or both. When seeded on matrices from TGF-β1-treated CAFs, SW480 and T47D cancer cells presented enhanced spreading, which is mirrored by the significantly altered cell shapes. HCT8/ E11 cells showed a trend towards enhanced cell spreading on matrices from TGF-β1-treated CAFs, and significant alterations on matrices from bFGF/TGF-β1-treated CAFs. SKBR3 cells displayed only limited, statistically non-significant alterations in cell spreading and factor shape. N.S.= non-significant (p>0.05); circles = outliers; squares = extremes.

The rate of cancer cell spreading was quantified by determining the factor shape of twenty cells on each matrix (Figure 9). This was not possible for the BT474 and CaCo-2 cell lines, as these grow in dense islands in which separate cells cannot be distinguished. SW480 and T47D cancer cells presented enhanced spreading when seeded on matrices from TGF-β1-treated CAFs, which is reflected by the significantly altered cell shapes (p=0.010 and p=0.046, respectively). There was a trend toward increased cell spreading when seeded on matrices of bFGF/TGF-β1-treated CAFs, though not statistically significant. HCT8/ E11 cells showed a trend towards enhanced cell spreading on matrices from TGF-β1-treated CAFs, and distinct alterations on matrices from bFGF/TGF-β1-treated CAFs (p<0.001). SKBR3 cells displayed only limited, statistically non-significant alterations in cell spreading and factor shape. Although some SKBR3 cells displayed a more irregular shape, the majority retained its epitheloid morphology (Figure 9D). Altogether, the results of these experiments indicate that TGF-β1-induced modulation of the tumor microenvironment affects cancer cell spreading but not adhesion.

## DISCUSSION

The ECM undergoes many alterations which support expansion and invasion of tumors [2, 13]. Such alterations are reflected in the stromal architecture of DCIS of the breast, since we recently observed that the presence of periductal myxoid stroma is a potential prognostic marker for increased recurrence risk in DCIS [11]. Myxoid stroma was strongly associated with reduced stromal decorin expression [11]. As decorin plays an important role in the assembly of collagen fibrils [12], decreased stromal decorin may contribute to the development of myxoid stroma. In this study, we identified TGF-β1 and bFGF as potent decorin suppressors in CAFs. In addition, TGF-β1 demonstrated to be an overall powerful ECM modulator, as it strongly upregulated type I collagen, versican and biglycan. Similar effects, though less pronounced, were observed by treating CAFs with bFGF or cancer-cell derived CM.

Expression of α-SMA was differentially regulated by TGF-β1 and bFGF: both cytokines downregulated decorin and upregulated versican, whereas α-SMA was induced by the former and suppressed by the latter, in accordance with previous studies [15, 16]. This might explain the previously reported lack of correlation between myxoid stroma and stromal α-SMA expression in DCIS, despite the distinct association between stromal architecture and decorin expression [11]. It would be of interest to determine if versican is a more robust CAF marker than α-SMA. As bFGF downregulates α-SMA but induces versican, some CAFs might be α-SMA-negative but versican-positive, depending on the predominant cytokine production by adjacent neoplastic cells. Nevertheless, secretome experiments show that nearly all breast and colorectal cancer cell lines induce both α-SMA, versican and biglycan in CAFs, which implies TGF-β1-like effects. Similar observations were reported by others. Co-cultures of CaCo-2 or HT29 cells with canine mammary stromal cells resulted in TGF-β1-induced versican expression [20]. Coulson-Thomas et al. observed that direct cell-cell contact of CaCo-2 or HCT116 with fibroblasts induced an increased expression of collagens and biglycan in the latter [21]. TGF-β1 and CM from pancreatic cancer cells synergistically suppressed decorin and lumican, and stimulated versican expression in pancreatic stellate cells [22]. CM of all colorectal cancer cell lines upregulated decorin expression, while CM of the majority of the breast cancer cell lines caused decorin suppression. We speculate that TGF-β1-induced decorin downregulation is counteracted by another, presently unknown, ligand in colorectal cancer CM. Despite this finding, stromal decorin seems to be consistently downregulated in different types of cancer compared with normal tissue, as demonstrated by Bozoky et al. [23]. Decrease of stromal decorin during colorectal and breast cancer progression has also been shown by others [9, 10].

Stromal biglycan expression was not related to stromal architecture, but a trend was noted for stromal versican expression, as myxoid DCIS displayed twice as often increased versican immunoreactivity. Various studies have demonstrated increased stromal versican expression in tumors compared with normal tissue, such as in cancer of prostate, breast, colon, pancreas and ovary [22, 24-28]. Versican formed part of a 74-gene profile that classified IDC and DCIS, and revealed to be upregulated in IDC [6]. Canavese et al. studied versican in a DCIS cohort without available outcome data [29]. Stromal versican expression defined a specific subtype of DCIS, characterized by high nuclear grade and often presenting comedonecrosis [29]. We speculate that the majority of DCIS with high periductal versican will also present myxoid stroma and reduced decorin expression, and patients with such lesions may be more likely to relapse. Stromal versican overexpression has been investigated in patients with node-negative invasive breast cancer and was associated with reduced relapse-free survival [30, 31]. In colorectal cancer, upregulated biglycan expression was associated with an increased risk of both lymph node and distant metastasis [32]. Future research on large patient cohorts is warranted to explore the value of stromal versican and biglycan expression as a prognostic marker for recurrence in DCIS.

In addition, we investigated the influence of decorin on cancer cell adhesion. Decorin effectively inhibited adhesion of both breast and colorectal cancer cells, although this was counteracted by type I collagen. According to Kenny et al., ovarian cancer cells preferentially adhere to and invade type I collagen [33]. As the mammary stroma contains more than just decorin and type I collagen, we attempted to mimic the *in vivo* situation by preparing CAF-derived matrices [18]. Consequently, matrices from TGF-β1-treated CAFs hold higher versican and biglycan levels and lower decorin levels than matrices from untreated CAFs. Nevertheless, no significant quantitative differences in adhesion were noted between the various matrices, although cancer cells displayed a distinct ‘track-like’ spreading pattern when seeded on matrices from TGF-β1-treated CAFs. We surmise that decorin is part of a stromal barrier, preventing cancer cell spreading and thus invasion. Consequently, neoplastic cells need to modulate the ECM composition to overcome this stromal barrier and to create an invasion-permissive stroma. Such modulations can exist of paracrine regulation of ECM protein expression, which includes downregulation of decorin expression and upregulation of versican, biglycan and type I collagen.

We are aware that other mechanisms may contribute as well [2]. In our study we omitted to address enzymatic remodeling of the stroma. Hawinkels et al. described that cancer cell derived TGF-β1 turns fibroblasts into α-SMA-positive CAFs that produce matrix metalloproteinases (MMP) [34]. Release of MMP in the stromal compartment could contribute to the breakdown of anti-adhesive proteoglycans like decorin. According to Imai et al., decorin contains cleavage sites for MMP-2 (gelatinase-A), MMP-3 (stromelysin-1) and MMP-7 (matrilysin) [35]. Gene expression profiling revealed that transition of in situ to invasive breast cancer was characterized by an increased expression of MMP in the stroma [4-6]. Other protease classes, suchs as cathepsins, may digest decorin as well [17, 36]. Proteolytic activity is also required to activate TGF-β1, since this cytokine is secreted as a latent molecule and sequestered by several ECM components. The ECM acts as a reservoir for cytokines, and decorin is able to bind TGF-β1 [37]. Proteolytic degradation of decorin results in release of this sequestered TGF-β1 [35].

TGF-β1 is an attractive candidate for targeted therapy, as this might prevent extensive stromal alterations during cancer progression. Lifetime exposure to a TGF-β antagonist protected mice against metastasis without adverse side effects [38]. According to Liu et al., TGF-β blockade in orthotopic mammary carcinoma mouse models significantly reduced tumor growth and metastasis [39]. Interestingly, this TGF-β blockade decreased the type I collagen content, which contributed to normalization of the tumor stroma and improved intratumoral penetration of therapeutics [39]. Further elucidation of ECM changes and their role in cancer progression is warranted. Naba et al. developed a proteomic strategy to determine the *in vivo* ECM composition of tumors and normal tissues [40]. Such a characterization of the ‘matrisome’ revealed that tumor-and stroma-derived ECM components differ among tumors with different metastatic potential [40]. This new strategy offers perspectives for future ECM analysis.

In conclusion, preinvasive tumoral lesions may shape the composition of the adjacent ECM through TGF-β1 release to secure an invasion-permissive microenvironment. Versican may be a more robust CAF-marker than α-SMA, although further research is warranted. Additional investigations on larger patient cohorts are required to elucidate the ability of altered stromal protein expression as prognostic marker in breast and colorectal cancer in general, and in DCIS of the breast in particular.

## METHODS

### Cell culture and conditioned medium

Telomerase-immortalized human colon cancer-associated fibroblasts (CAFs) were maintained in Dulbecco's minimal essential medium (DMEM), supplemented with 10% fetal calf serum (FCS), 100 U/ml penicillin, 100 μg/ml streptomycin and 2.5 μg/ ml fungizone (Life Technologies, Ghent, Belgium). Isolation and characterization of the CAFs was previously described [41]. Experiments were performed with human breast cancer cell lines BT474, MCF-7, MDA-MB-231, MDA-MB-453, SKBR3 and T47D, and human colorectal cancer cell lines CaCo-2, HCT8/E11, HT-29 and SW480. All cell lines were grown in DMEM supplemented with 10% FCS, fungizone and antibiotics, except CaCo-2 cells (additionally supplemented with 5 μg/ml transferrin and 1% MEM-NEAA or non-essential amino acids solution; Life Technologies), and HT-29 (McCoy's medium supplemented with antibiotics, fungizone and 10% FCS; Life Technologies). Serum-free 10x concentrated conditioned medium (CM) derived from 3×107 cancer cells was obtained after 24h of incubation and prepared as previously described [41]. HEK-293-EBNA-*DCN* cells (kindly provided by dr. Ake Oldberg, Lund University, Sweden) were cultured in DMEM supplemented with 10% FCS, antibiotics and fungizone, and 100 μg/ml hygromycin B (Life Technologies) for selection [42]. CM from HEK-293-EBNA-*DCN* cells containing His-tagged human recombinant decorin was collected after 48h of incubation and 10x concentrated with Centricon Plus-70 centrifugal filters (Merck Millipore, Overijse, Belgium). Concentrated CM was stored at −20°C until decorin purification.

### Reagents and experimental set-up

The following human recombinant proteins were used: bFGF (PeproTech EC, London, UK), EGF, TGF-α (both by Sigma-Aldrich, St Louis, MO, USA), IGF-1, IL-6, IL-8, NRG-β1, OSM, TGF-β1 and TNF-α1 (all by R&D Systems, Minneapolis, MN, USA). CAFs were grown in T-25 tissue culture flasks and treatment with cytokines (in the presence of 10% FCS) or CM (in the presence of 5% FCS) started when 95% confluence was reached. Medium was changed every 48h. After 6 days of treatment, cell lysates or RNA samples were prepared.

### Cell lysates, SDS-PAGE and Western blots

CAFs were harvested in Laemmli lysis buffer (0.125 M Tris-HCl, 10% glycerol, 2.3% sodium dodecyl sulfate (SDS), pH 6.8). Lysates were suspended in reducing sample buffer (0.5 M Tris-HCl, 40% glycerol, 9.2% SDS, 4.5% 2-mercaptoethanol, 0.011% bromophenolblue, pH 6.8) and boiled at 95°C for 5 minutes. Equal protein amounts were separated on 8% polyacrylamide gels, transferred to nitrocellulose membranes (Bio-Rad, Hercules, CA, USA), blocked in 5% nonfat milk in phosphate-buffered saline (PBS) with 0.5% Tween-20 (Sigma-Aldrich), and immunostained. The following antibodies were used: anti-α Smooth Muscle Actin (SMA, clone 1A4, Sigma-Aldrich), anti-biglycan (H-150, SantaCruzBiotechnology, Santa Cruz, CA, USA), anti-decorin (clone 115402, R&D Systems), anti-versican (H-56, SantaCruz Biotechnology). Detection was performed by horseradish peroxidase-conjugated secondary antibodies (all GE Healthcare, Pittsburgh, PA, USA) and chemiluminescence (Pierce ECL Western blotting substrate, Thermo Scientific, Rockford, IL, USA) according to the manufacturer's instructions. Membranes were stripped with ReBlot Plus mild antibody stripping solution (Merck Millipore) and reprobed with anti-α-tubulin (clone B-5-1-2, Sigma-Aldrich) as a loading control. Quantification of protein expression was performed with ImageJ software (NIH, MD, USA).

### RNA-isolation and RT-qPCR analysis

CAFs were trypsinized and cell pellets were washed twice with RNase-free water. RNA was isolated with the miRNeasy kit (Qiagen, Venlo, The Netherlands), cDNA synthesis was performed with the iScript cDNA synthesis kit (Bio-Rad), and RT-qPCR analysis was performed on the MyiQ RT-PCR detection system (Bio-Rad) by using Mesa Green qPCR MasterMix Plus (Eurogentec, Seraing, Belgium), in accordance with the manufacturer's instructions. A preliminary experiment was conducted to identify three appropriate reference genes (*TBP*, *YWHAZ*, *GAPDH*) out of a set of ten genes by using qBASE+ software (Biogazelle, Zwijnaarde, Belgium). Primers (Biolegio, Nijmegen, The Netherlands) are displayed in Table 1. All primers were blasted in Primer Blast (NCBI).

**Table 1:** Forward and reverse primer sequences for RT-qPCR analysis of mRNA expression

Gene	Forward primer 5′−3′	Reverse primer 5′−3′
*ACAN*	TGCATTCCACGAAGCTAACCT	GACGCCTCGCCTTCTTGA
*ACTA2*	GGAATGGGACAAAAAGACAGCTA	CGGGTACTTCAGGGTCAGGAT
*ASPN*	TCACTTTATGGTCTGATCCTGAACA	CTTCGCAACTTCTTTGTGGTTAGA
*BGN*	ACAAACTGCCCAGGAGTGAGTAG	GCCGTCCGCACGTCTATCT
*COL1A1*	CAACCTCAAGAAGTCCCTGC	AGGTGAATCGACTGTTGCCT
*DCN*	TCAATGGACTGAACCAGATGA	CCTTGAGGAATGCTGGTGAT
*GAPDH*	TGCACCACCAACTGCTTAGC	GGCATGGACTGTGGTCATGAG
*FMOD*	CAGTATGAAGATGACCCTCATTGG	TAAGGGTCATAGGGATCGTAGTAGGT
*LUM*	CTTCAATCAGATAGCCAGACTGC	AGCCAGTTCGTTGTGAGATAAAC
*TBP*	CACGAACCACGGCACTGATT	TTTTCTTGCTGCCAGTCTGGAC
*VCAN*	CAAGCATCCTGTCTCACGAA	CAACGGAAGTCATGCTCAAA
*YWHAZ*	ACTTTTGGTACATTGTGGCTTCAA	CCGCCAGGACAAACCAGTAT

### Immunohistochemistry (IHC)

Twenty specimens were selected out of a DCIS cohort diagnosed at Ghent University Hospital between 1 January 2007 and 31 December 2011, with approval of the local ethics committee. Selection was based on stromal architecture and availability of formalin-fixed, paraffin-embedded (FFPE) tissue blocks. IHC for decorin and α-SMA has previously been performed [11]. IHC for versican (dilution 1/25, HPA004726, Atlas Antibodies, Stockholm, Sweden) and biglycan (dilution 1/200, HPA003157, Atlas Antibodies) was performed on 3.5 μm FFPE tissue sections, using a Ventana Automated Slide Stainer (Benchmark XT, Ventana Medical Systems, AZ, USA). Heat-induced epitope retrieval was carried out using CC2 (Ventana Medical Systems). Visualization was achieved with the ultraViewTM Universal DAB Detection Kit (Ventana Medical Systems). Optimal dilutions and conditions were initially determined by IHC on placenta tissue (biglycan) and cerebral cortex (versican). Stromal expression was scored semi-quantitatively as low (or low to moderate) or high. Versican IHC of one DCIS case was not assessable because of tissue exhaustion; nineteen DCIS remained for evaluation.

### Purification of decorin

His-tagged human recombinant decorin was purified from 10x concentrated CM from HEK-293-EBNA-*DCN* cells. CM was adjusted to 20 mM Tris and 300 mM NaCl (pH 8.0) with stock solutions and incubated with Ni2+- NTA agarose beads (Qiagen) at 4°C for 1h with agitation. Beads were collected by centrifugation and washed five times (mild centrifugation at 4°C) with wash buffer (20 mM Tris and 300 mM NaCl). Supernatant was disgarded. Recombinant decorin was eluted by adding elution buffer (20 mM Tris, 300 mM NaCl, 300 mM imidazole, pH 7.5). Supernatans was collected (mild centrifugation at 4°C) and 10x concentrated with 3K centrifugal filters (Merck Millipore). The concentrate was then diluted in excess PBS and concentrated again to wash away remaining imidazole. Decorin concentration was determined by using a spectrophotometer. Samples were passed through a 0.2μm Whatman filter before storage at −20° until further use.

### Coating assays

For single reagent assays, 96-well plates were incubated overnight at 37°C with type I collagen or purified decorin in PBS. For combined coating assays, 96-well plates were incubated 1h with type I collagen (rat tail, SantaCruz) in PBS and washed once with PBS, before overnight incubation with purified decorin in PBS at 37°C. Wells were washed once with PBS to remove excess of unbound proteins, before seeding cancer cells onto the coatings (10.000 cells/well). After 24h of incubation at 37° in a 5% CO_2_ incubator, SRB and MTT assays were performed.

### MTT

Culture medium of 96-well plates was replaced by 100 μl culture medium containing 1mg/ml MTT. Following 2h incubation at 37°C, MTT-containing medium was removed and 150 μl of dimethylsulfoxide (DMSO) was added to dissolve formazan crystals. Absorbance was measured at 570 nm with a spectrophotometer (Paradigm, Molecular Devices, USA).

### SRB

Culture medium of 96-well plates was removed and cells were fixed with 50 μl 50% trichloroacetic acid solution for 1h. Plates were washed 5 times with water. After air drying, 50 μl of 0.4% SRB in 1% acetic acid solution was added to each well and incubated for 30 minutes at 4°C. After SRB removal, plates were washed 5 times with 1% acetic acid before air drying. Bound SRB was solubilized with 200 μl 10 mM Tris buffer (pH 10.5). Absorbance was measured at 570 nm with a spectrophotometer (Paradigm, Molecular Devices, USA).

### Production of CAF-derived matrices

CAF-derived matrices were generated according to an adaptation of the protocol of Castello-Cros et al. for preparation of fibroblast-derived matrices in 96-well (10.000 cells/well) and 6-well (300.000 cells/well) culture plates [18]. Cell spreading was quantified by assessing the factor shape (i.e. inverse circularity or P2/4πA) in Adobe Photoshop CX5 software (Adobe Systems Incorporated, San Jose, CA, USA).

### Cell-electrode impedance attachment assay

Real-time monitoring of cell adhesion was assessed by an electrical impedance assay with an xCELLigence RTCA SP real-time cell-sensing device (ACEA Biosciences, San Diego, CA, USA). E-plates (ACEA Biosciences) were coated in quadruplicate with 10 μg/ml decorin, 10 μg/ml type I collagen or both (as described under coating assays). CaCo-2 and BT474 cells (10.000/ well) were seeded into coated E-plates and impedance was measured every 3 minutes for 24h. All assays were performed twice. Adhesion was expressed as the cell index, i.e. the change in electrical impedance at each time point (mean ± SD). Slopes for each curve were calculated.

### Statistics

Statistical analysis was performed in IBM SPSS 21.0 (SPSS, Chicago, IL, USA). Differences in cell adhesion on coatings were assessed by one-way ANOVA for SRB and MTT assays, and by Mann-Whitney U test on calculated slopes for electrical impedance assays. Differences in IHC staining between sclerotic and myxoid DCIS were analyzed using Fisher's exact test. Differences in factor shape were assessed using Mann-Whitney U test. All mentioned tests were two-sided, and p<0.05 was considered to be statistically significant. Power of IHC was calculated in SAS Power & Sample Size (SAS Institute, Cary, NC, USA).

## Abbreviations

ACAN: aggrecan
ACTA2: alpha smooth muscle actin
ASPN: asporin
bFGF: basic fibroblast growth factor
BGN: biglycan
CAF: cancer-associated fibroblast
CM: conditioned medium
COL1A1: type 1 collagen alpha-1
DCIS: ductal carcinoma in situ
DCN: decorin
DMEM: Dulbecco's minimal essential medium
DMSO: dimethylsulfoxide
ECM: extracellular matrix
EGF: epidermal growth factor
FCS: fetal calf serum
FFPE: formalin-fixed, paraffin-embedded
FMOD: fibromodulin
IDC: invasive ductal carcinoma
IGF: insulin-like growth factor
IHC: immunohistochemistry
IL: interleukin
LUM: Lumican
MMP: matrix metalloproteinase
MTT: 3-(4,5-dimethylthiazol-2yl)-5,5-diphenyltetrazolium bromide
NEAA: non-essential amino acids
NS: non-significant
NRG: neuregulin
OSM: oncostatin-M
PBS: phosphate-buffered saline
RT-qPCR: real time quantitative polymerase chain reaction
SD: standard deviation
SDS: sodium dodecyl sulfate
SLRP: small leucin-rich proteoglycans
SMA: smooth muscle actin
SRB: sulforhodamine B
TGF: transforming growth factor
TNF: tumor necrosis factor
VCAN: versican
